# Ultrafast Time-Stretch Optical Coherence Tomography Using Reservoir Computing for Fourier-Free Signal Processing

**DOI:** 10.3390/s25123738

**Published:** 2025-06-15

**Authors:** Weiqing Liao, Tianxiang Luan, Yuanli Yue, Chao Wang

**Affiliations:** 1 Photonics Information Innovation Center and Hebei Provincial Center for Optical Sensing Innovations, College of Physics Science & Technology, Hebei University, Baoding 071002, China; wl258@kent.ac.uk; 2 School of Engineering, University of Kent, Canterbury CT2 7NT, UK; tl436@kent.ac.uk (T.L.); yy282@kent.ac.uk (Y.Y.)

**Keywords:** optical coherence tomography, reservoir computing, photonic time stretch

## Abstract

Swept-source optical coherence tomography (SS-OCT) is a widely used imaging technique, particularly in medical diagnostics, due to its ability to provide high-resolution cross-sectional images. However, one of the main challenges in SS-OCT systems is the nonlinearity in wavelength sweeping, which leads to degraded depth resolution after Fourier transform. Correcting for this nonlinearity typically requires complex re-sampling and chirp compensation methods. In this paper, we introduce the first ultrafast time-stretch optical coherence tomography (TS-OCT) system that utilizes reservoir computing (RC) to perform direct temporal signal analysis without relying on Fourier transform techniques. By focusing solely on the temporal characteristics of the interference signal, regardless of frequency chirp, we demonstrate a more efficient solution to address the nonlinear wavelength sweeping issue. By leveraging the dynamic temporal processing capabilities of RC, the proposed system effectively bypasses the challenges faced by Fourier analysis, maintaining high-resolution depth measurement without being affected by chirp-introduced spectral broadening. The system operates by categorizing the interference signals generated by variations in sample position. This classification-based approach simplifies the data processing pipeline. We developed an RC-based model to interpret the temporal patterns in the interferometric signals, achieving high classification accuracy. A proof-of-the-concept experiment demonstrated that this method allows for precise depth resolution, independent of system chirp. With an A-scan rate of 50 MHz, the classification model yielded 100% accuracy with a root mean square error (RMSE) of 0.2416. This approach offers a robust alternative to Fourier-based analysis, particularly in systems prone to nonlinearities during signal acquisition.

## 1. Introduction

Since its introduction by D. Huang et al. in 1991 [1], optical coherence tomography (OCT) has garnered significant attention due to its non-invasive nature and high-resolution imaging capabilities, making it widely applicable in fields such as biology and industry [2,3,4]. Building on this foundation, a research team from MIT described Swept Source OCT (SS-OCT) in 1994 [5,6], followed by a team from the University of Vienna in 1995 [7,8], which demonstrated the use of tunable lasers and spectrometer-based interferometry to measure intraocular distances. SS-OCT seeks to combine some of the advantages of standard Time Domain (TD) and Spectral Domain (SD) OCT. In SS-OCT, the spectral components are encoded not by spatial separation but rather by temporal encoding, where the spectrum is either filtered or generated in single continuous frequency steps, subsequently reconstructed prior to Fourier transformation. By utilizing a frequency-scanning light source (i.e., a frequency-swept laser), the optical setup becomes simpler compared with SD-OCT, but the challenge of scanning is effectively shifted from the TD-OCT reference arm to the SS-OCT light source. Swept laser sources can achieve very narrow instantaneous bandwidths (linewidth) at extremely high frequencies [9].

The sweep rate of traditional SS-OCT systems is constrained by the laser repetition rate, leading to the development of various laser technologies aimed at improving the A-scan rate [10,11,12]. Conventional swept lasers incorporate a tunable filter placed in the external cavity of the laser, which controls the wavelength of light allowed to oscillate within the cavity. By altering the properties of the filter, the laser can gradually change its output wavelength, enabling wavelength sweeping. However, the scan rate is limited to the kilohertz range, as the laser must regenerate from spontaneous emission with each round trip. In 2011, to further enhance SS-OCT scanning speed, researchers integrated widely tunable micro-electromechanical systems (MEMSs) vertical-cavity surface-emitting lasers (VCSELs) with SS-OCT, achieving a significant increase in sweep rates from the kilohertz to the megahertz range [13]. The MEMS-VCSEL-based SS-OCT leverages the high tuning speed and precision of MEMS technology and VCSELs to enable rapid, stable, and high-resolution three-dimensional imaging, particularly suitable for dynamic and in vivo biological imaging. However, mechanical tunable filters used in swept lasers are limited by inertia, resulting in tuning speeds typically in the kilohertz range, which can be alleviated by adopting non-mechanical or passive scanning methods.

In recent years, Time-Stretch Dispersive Fourier Transform (TS-DFT) has been proven to be a promising method for increasing OCT scanning speed [14]. In TS-DFT, a dispersive medium (e.g., a dispersive optical fiber) is used to temporally stretch the incoming short-pulse light. Due to chromatic dispersion, different wavelength components within the light pulse propagate at different speeds, causing the pulse to stretch during transmission. This means that the time distribution of the wavelength components within the pulse is expanded, making them temporally separated. The resulting chirped pulse sequence functions as a passive swept source, enabling an axial scanning rate in SS-OCT equivalent to the laser’s pulse repetition rate [15,16,17]. However, the wavelength-to-time mapping in a time-stretch system is typically nonlinear due to the inherent high-order dispersion in optical fibers [18]. Frequency chirp is introduced to the interference waveform due to nonlinear mapping, leading to degradation in OCT depth resolution and potential image distortion or blurring after Fourier transformation. Alternative digital post-processing techniques have been proposed, including resampling algorithms such as linear interpolation and polynomial fitting [19,20], though these methods exhibit limited accuracy when addressing complex nonlinearities.

Existing methods primarily focus on correcting nonlinear sweeping and mitigating spectrum broadening after Fast Fourier Transform (FFT) [21,22]. However, these approaches suffer from high computing costs and increased processing time. This is particularly challenging in TS-OCT systems as the normal sampling rate is extreme (tens of GSa/s). Compressive sensing has been successfully applied in TS-OCT to reduce the data sampling rate and to estimate the frequency information of the interference waveform without Fourier transform analysis [23], but it still suffers from chirp-induced spectrum broadening.

On the other hand, direct time-domain signal analysis without relying on computationally intensive Fourier transform offers a promising alternative demodulation solution for a time-varying system [24]. The temporal waveform of the OCT signal changes inevitably with varying axial sample distances. Therefore, based on temporal signal analysis, precise distance measurement can be achieved by distinguishing waveforms corresponding to different distances through sequence data classification and time series prediction methods. Current approaches often rely on Recurrent Neural Networks (RNNs) [25] and Long Short-Term Memory (LSTM) networks [26] for modeling temporal dependencies in sequential data; however, they suffer from the vanishing gradient problem, which limits their ability to capture long-term dependencies. LSTMs were developed to overcome this issue by introducing gating mechanisms that enable them to retain information over longer sequences. While effective, LSTM networks require extensive training through backpropagation, which is computationally intensive and slow, especially for tasks involving complex, long-term dependencies. Reservoir computing (RC) has been developed as a special variant of RNN [27,28]. RC has gained recognition as a highly efficient solution for complex time-series analysis and classification tasks [29,30,31]. RC stands out for its remarkable capability in handling nonlinear temporal dependencies. Unlike conventional RNN models, RC only requires training of the output weights, thus significantly reducing the time and computational resources needed for model optimization, while still maintaining competitive accuracy. RC is particularly well-suited for tasks involving time-series data, as the reservoir’s recurrent structure allows it to capture both short-term and long-term temporal dependencies. For example, in applications such as optical coherence tomography (OCT) or frequency-modulated continuous-wave (FMCW) LiDAR systems [32], where the input signals exhibit complex nonlinear dynamics, RC can quickly classify and process the time-series signals to produce accurate results.

Moreover, RC’s simplicity in structure and computation enables it to outperform other methods in terms of speed and efficiency. For instance, while traditional RNN models suffer from backpropagation through time (BPTT) with a complexity of $O(N^{2})$, RC reduces the overall computational complexity to O(N), making it a more scalable choice for real-time applications. This advantage is especially prominent in high-frequency temporal signal analysis, where the reduced computational load of RC allows for faster processing and real-time implementation without compromising performance. As shown in Table 1, the RC model demonstrates significant advantages over RNN and LSTM networks, particularly in terms of computational efficiency, processing speed, and scalability.

In this study, we propose and validate an innovative Fourier-free TS-OCT system that employs an RC model for highly efficient time-domain signal processing without relying on Fourier transformation. By fully utilizing the temporal characteristics of the interference signal with the presence of nonlinear sweeping, we achieve precise depth measurements in OCT via the classification of temporal waveforms [33]. Unlike traditional Fourier-based TS-OCT systems, our novel system avoids complex signal linearization steps and spectrum broadening issues. In addition, RC is much less computationally intensive than fast Fourier transform (FFT) [29]. For instance, while the computational complexity of FFT for a dataset with a sample size *N* is $N\log_{2}^{N}$, the complexity of RC is merely *N*. Therefore, through the introduction of RC technology, the system can quickly and accurately extract depth information from time-domain signals while reducing the computational burden of processing.

## 2. Principles

### 2.1. Fourier-Free TS-OCT System

**Figure 1:**
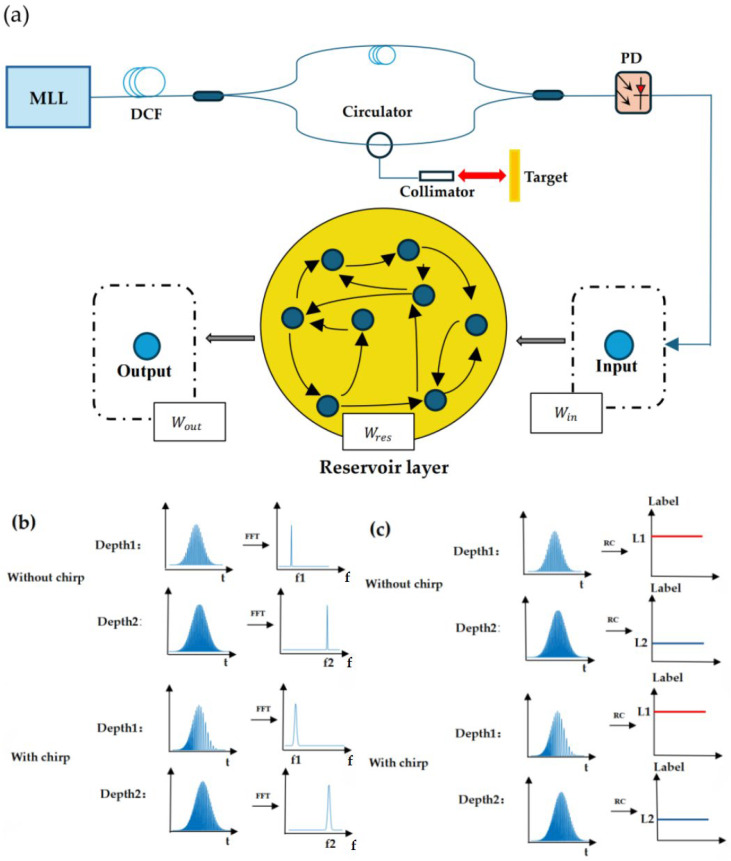
(**a**) Schematic diagram of the proposed Fourier-free TS-OCT system employing an RC module for temporal signal analysis. (**b**) Results of traditional Fourier analysis: nonlinear wavelength sweeping introduces frequency chirp and broadens the FFT peaks, hence poor depth resolution. (**c**) RC-based temporal signal analysis directly determines the depth information without being affected by the frequency chirp.

The experiment was set up based on the TS-OCT system design shown in Figure 1a. This proof-of-concept system uses a passively mode-locked fiber laser (MLL, Calmar Mendocino FP laser) as the light source, producing a train of ultrashort pulses with a duration of 800 femtoseconds (fs) and a spectral bandwidth of 12 nm. The laser operates at a repetition frequency of 50 MHz, generating high-frequency pulse trains for rapid imaging. The experiment was set up based on the TS-OCT system design shown in Figure 1a. This proof-of-concept system uses a passively mode-locked fiber laser (MLL, Calmar Mendocino FP laser) as the light source, producing a train of ultrashort pulses with a duration of 800 femtoseconds (fs) and a spectral bandwidth of 12 nm. The laser operates at a repetition frequency of 50 MHz, generating high-frequency pulse trains for rapid imaging. Initially, the ultrashort pulses propagate through a dispersion-compensating fiber (DCF) exhibiting a total chromatic dispersion amount of −1.04 ns/nm, which stretches each 800 fs pulse to 8 ns. This time stretch process effectively creates a broadband, passive wavelength-swept optical source. The resulting wavelength-sweeping effect is essential for high-throughput imaging in OCT.

Following this, the wavelength-swept light is split by a 50:50 coupler and directed into a Mach–Zehnder Interferometer (MZI) structure, which forms the core of the OCT system. The MZI has two arms: the reference arm and the measurement arm. In the reference arm, a 100-m segment of optical fiber is added as an extra dispersion element to enhance nonlinear effects in the interference waveforms [33], which improves the RC model’s accuracy in depth measurements by creating a more complex temporal pattern in the reference signal. In addition, this introduced additional dispersion creates a frequency-chirped interference signal that enables testing and validating the RC model’s chirp-tolerant capabilities. Meanwhile, in the measurement arm, the light is routed through an optical circulator and directed to a collimator. The collimated beam is then reflected by an axially movable mirror, enabling depth variation within the measurement path. The backscattered light from the mirror is recoupled through the circulator and directed back toward the beam splitter.

The light from the reference and measurement arms interferes with the beam splitter, producing time-domain interference fringes. This interference pattern reflects the depth-resolved information required for OCT imaging. The interference signal is then detected by a high-speed photodetector (PD), capable of capturing the high-frequency fringes produced by the TS-OCT system. A real-time oscilloscope (Tektronix DSA73304D) records the signal for further analysis. The oscilloscope enables high-resolution temporal recording, ensuring that the fine details of the interference signal are preserved. The signal acquired by the oscilloscope is subsequently processed using the RC model. The fundamental principle of RC is to project the input data into a high-dimensional state space via a fixed, dynamic reservoir. Acting as a nonlinear dynamical system, the reservoir maps sequential input data into a set of intricate internal states that effectively encapsulate temporal dependencies. A comprehensive explanation of the RC methodology will be provided in Section 2.2.

In a chirp-free system, where the dispersion-compensating fiber (DCF) does not introduce higher-order dispersion, a uniform interference waveform with a single carrier frequency is generated. This results in a clean and stable signal, enabling accurate and high-resolution depth measurements through Fourier transformation, as illustrated in Figure 1b. However, when nonlinear wavelength sweeping occurs due to higher-order dispersion effects in long DCFs, the interference waveform becomes frequency-chirped. This chirping introduces broadened peaks in the FFT output, which significantly degrades the axial resolution of OCT imaging and reduces depth measurement accuracy.

To address this issue, our proposed system bypasses FFT analysis altogether, instead employing an RC model for real-time distance measurement by directly analyzing the time-domain signals. This Fourier-free approach allows the system to maintain high accuracy without needing to compensate for frequency chirp. By classifying time-domain patterns, the RC model provides output labels that correspond to specific depths, effectively mapping the temporal signal features directly to depth information, as shown in Figure 1c. In Figure 1c, the output labels from the RC model represent the axial depths of the sample, where the blue and red lines indicate different depths, with the red line corresponding to shallower depths and the blue line representing deeper regions. This method eliminates the need for re-sampling or chirp compensation, making the system highly efficient and robust against the distortions typically introduced by nonlinear sweeping. As a result, this RC-assisted TS-OCT system offers a streamlined solution for high-resolution OCT imaging, delivering reliable depth measurements even in the presence of chirp.

### 2.2. RC Approach for Temporal Signal Processing

Traditional optical coherence tomography systems typically require fast FFT to obtain axial depth information. However, the proposed method in this study, based on direct time-domain signal analysis using an RC model, enables a direct classification of interference waveforms from different depths. This approach allows for precise depth detection, with the target depth information determined from the classification results, regardless of the presence of frequency chirp, as shown in Figure 1c. Unlike FFT-based methods, which face challenges due to frequency linearization, increasing the system’s complexity, the proposed RC-based method avoids such issues, simplifying the depth detection process.

To process the acquired signal, the RC model utilizes a structured approach to map and analyze sequential data. The architecture of RC is designed to capture the temporal dynamics of the input efficiently while minimizing computational overhead. This is achieved through the interaction of its key components: the input layer, reservoir layer, and output layer.

The input layer projects the input sequence *u*(*t*) into the reservoir, using a randomly initialized input weight matrix $W_{in}$. The reservoir layer consists of interconnected units whose connections are defined by the reservoir weight matrix $W_{res}$, which is also randomly initialized but fixed after initialization. The input and reservoir weights are not trained during the learning phase, making RC highly efficient in terms of computational complexity.

At each time step t, the state of the reservoir is updated based on the input signal and the previous state of the reservoir. This is expressed mathematically by the following update equation:

(1) $$s\left(t+1\right)=f(W_{in}u\left(t\right)+W_{res}s\left(t\right))$$

where $s\left(t\right)$ represents the state of the reservoir at time *t*, $W_{in}$ is the input weight matrix, $W_{res}$ is the internal reservoir connection matrix, and *f*(·) is a nonlinear activation function, typically chosen as the hyperbolic tangent $f\left(x\right)=\tanh\left(x\right)$.

The reservoir layer’s behavior is governed by its echo state property (ESP), which ensures that its internal states asymptotically depend on the input sequence, rather than on initial conditions. To maintain stability, the spectral radius $\rho \left(W_{res}\right)$ of the reservoir matrix, defined as the largest absolute value among its eigenvalues, is constrained to be less than 1. For example, if the eigenvalues of $W_{res}$ are {0.5, −0.8, 1.2}, then the spectral radius $\rho \left(W_{res}\right)$ = 1.2. By ensuring $\rho (W_{res})<1$, the reservoir achieves stability, allowing its states to converge over time. This guarantees that the influence of past inputs decays over time, allowing the reservoir to focus on the most recent inputs. Additionally, the past inputs, referring to signals from earlier time steps such as *u*(*t* − 5), *u*(*t* − 4), decay over time due to the reservoir’s dynamics. This decay ensures that the influence of these earlier signals diminishes, allowing the reservoir to focus on more recent inputs, such as *u*(*t*), *u*(*t* − 1). By constraining the spectral radius $\rho \left(W_{res}\right)$ to be less than 1, the reservoir achieves this balance, ensuring stability and relevance to the current input sequence.

Unlike traditional recurrent neural networks, only the output layer weights in RC are trained, which significantly reduces computational complexity. The output of the reservoir system is computed as a linear combination of the reservoir states, as follows:

(2) $$\hat{y}\left(t\right)=W_{out}s\left(t\right)$$

where $W_{out}$ is the output weight matrix and $\hat{y}\left(t\right)$ is the predicted output at time t.

The training process involves adjusting the output weights $W_{out}$ by minimizing the error between the predicted outputs and the target outputs. This is typically done using ridge regression with a regularization term to prevent overfitting. The output weight matrix is computed as

(3) $$W_{out}={\left(S^{T}S+\lambda I\right)}^{-1}S^{T}Y$$

where S is the matrix of reservoir states, $S^{T}$ is its transpose, Y is the matrix of target outputs, I is the identity matrix, and λ is the regularization parameter. The parameter λ controls the complexity of the model by adding a penalty term to the magnitude of the weights, helping prevent overfitting.

Once the output weights $W_{out}$ have been trained, the RC model can be used for prediction. For any new input sequence, the reservoir states are updated, and the output is calculated using the trained output weights. This simple linear combination of the reservoir states makes RC highly efficient for real-time processing of time-series data, such as in dynamic systems with complex temporal dependencies.

The experiments were conducted on a system equipped with a 13th Gen Intel(R) Core (TM) i7-13650HX CPU (Intel Corporation, Santa Clara, CA, USA) operating at 2.60 GHz, 16 GB of RAM, and an NVIDIA GeForce RTX 4060 Laptop GPU (NVIDIA Corporation, Santa Clara, CA, USA), using Python 3.12 (Python Software Foundation, Beaverton, OR, USA).

## 3. Results

### 3.1. TS-OCT Signal Analysis

**Figure 2:**
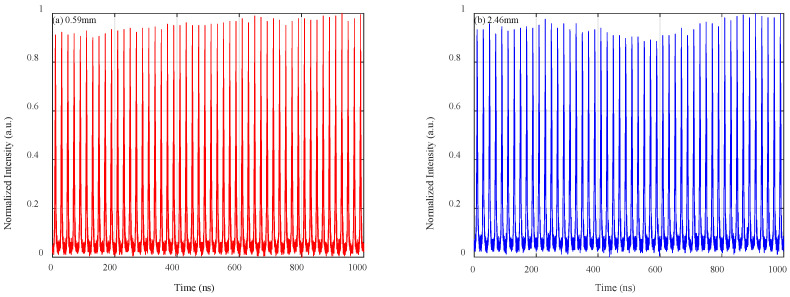

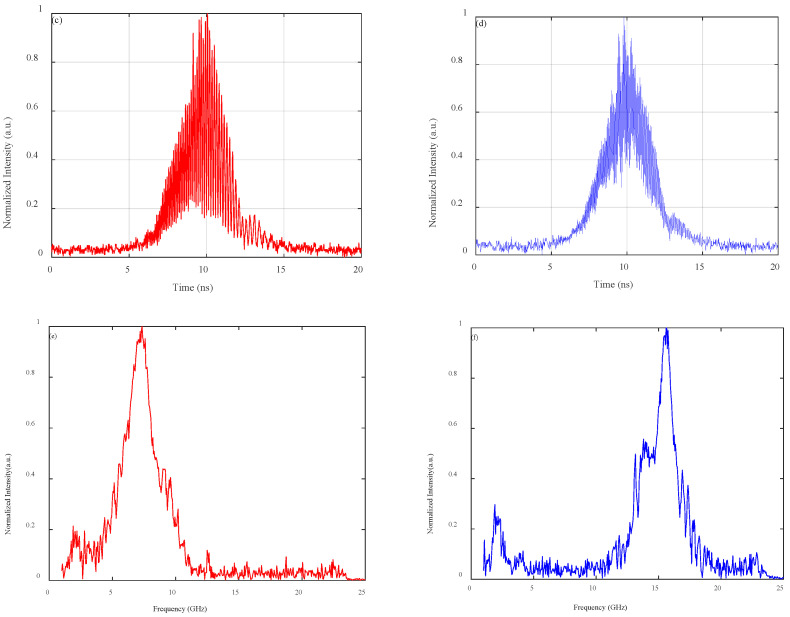
The measured 50 consecutive interference signal waveforms corresponding to a target depth of (**a**) 0.59 mm and (**b**) 2.46 mm. (**c**,**d**) are zoomed-in signals illustrating different temporal features within one single scanning period. To further investigate the impact of the chirp in the system, Fourier transforms were applied to the zoomed-in signals in (**c**,**d**), and the resulting frequency spectra are shown in (**e**,**f**).

Using the Fourier-free TS-OCT system mentioned earlier, 50 consecutive cycles of interference signals were captured by the oscilloscope within a 1 µs duration. These signals correspond to optical path lengths of 0.59 mm and 2.46 mm, as illustrated in Figure 2a,b, respectively. These time-domain interference signals represent different imaging depths and highlight the system’s capability to capture depth information at a refresh rate of 50 MHz in real time. A closer look at the interference waveforms within a single pulse period is provided in Figure 2c,d, which clearly demonstrate significant frequency chirping effects within each interference signal. This chirping is indicative of pronounced wavelength dispersion caused by the DCF and the extra optical fiber in the reference arm in the TS-OCT system.

To further analyze the impact of this chirp, a Fourier transform was applied to the interference patterns in Figure 2c,d, with the resulting frequency spectra shown in Figure 2e,f. These spectra reveal substantial broadening, which confirms the presence of high-order dispersion in the system [34]. This spectral broadening directly affects the system’s axial resolution, as the frequency components become less distinct, leading to degraded depth precision. These observations emphasize the challenges posed by nonlinear sweeping in achieving high-resolution OCT imaging and underline the necessity of a chirp-tolerant approach, such as the proposed reservoir computing (RC) model, which enables direct depth measurement without relying on Fourier analysis.

The collected interference signals were divided into two datasets: a training set and a test set. The training set was used to teach the reservoir computing (RC) model to recognize specific temporal patterns within the time-domain signals, while the test set was reserved for evaluating the model’s classification accuracy and generalization capabilities. By processing these datasets, the RC model learns to identify depth information from temporal features directly as a classification task, without requiring Fourier transform-based analysis. In the following sections, we detail how the waveform recognition-based RC model processes these datasets and assess the model’s performance in accurate, real-time signal classification, highlighting its potential in high-throughput TS-OCT imaging applications.

### 3.2. Overcoming Challenges of Fourier Analysis in Nonlinear TS-OCT Using RC

Using the RC model for time-domain signal classification, the proposed system achieves depth measurements directly from the interference signal, bypassing the need for Fourier analysis. This novel approach validates the effectiveness of Fourier-free signal processing in TS-OCT, demonstrating that the RC model can accurately interpret depth information from the temporal characteristics of the interference waveform alone. The experimental setup combines dispersion-compensating fiber (DCF), an MZI structure, and high-speed detection, providing a robust framework for depth imaging even in the presence of significant dispersion and frequency chirping. This configuration highlights the RC model’s potential in enhancing OCT imaging data processing efficiency and resolution without the typical computational burden of Fourier-based analysis.

In particular, the RC model derives distance information by classifying the intermediate frequency patterns associated with varying depths in the MZI structure. Specifically, we implemented the RC model using an Echo State Network (ESN) architecture [35], which offers a highly efficient method for processing sequential data. The reservoir size was set to 1000 units to balance complexity with computational efficiency, and the reservoir weight matrix was designed with a spectral radius of 0.8 to ensure stability while retaining temporal memory. An input scaling factor of 0.2 adjusts the sensitivity of the reservoir to incoming signals, while a connection sparsity of 0.3 maintains a manageable level of connectivity among reservoir units, reducing computational costs. To improve generalization, a regularization parameter of 500 was applied during training.

**Figure 3:**
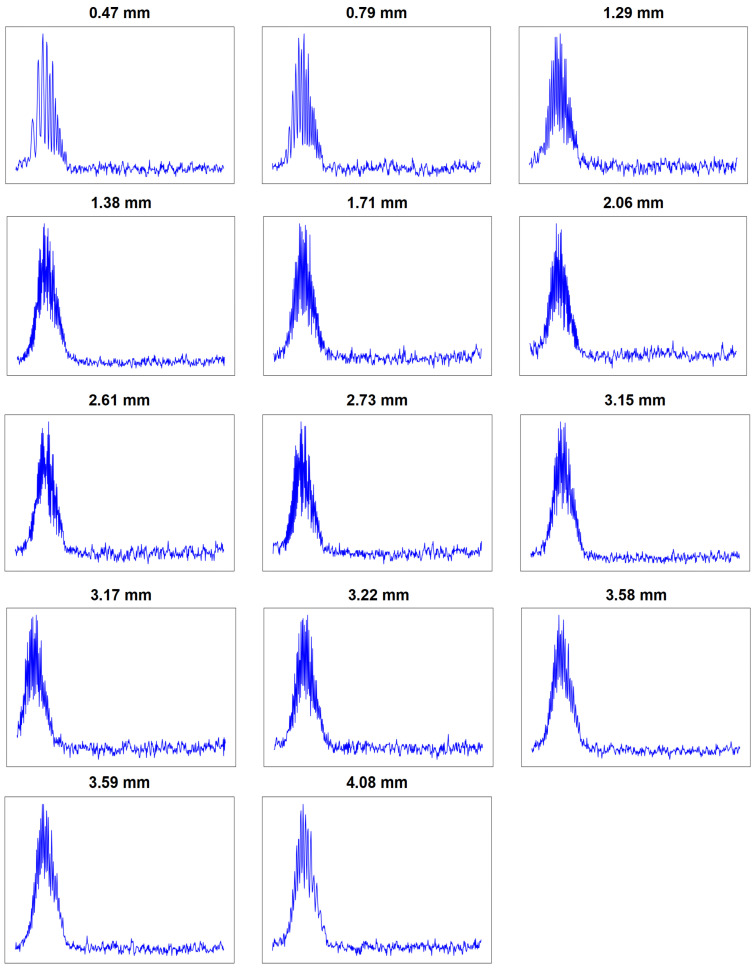
The measured interference signals corresponding to different axial distance differences.

To construct the training dataset, interference waveforms were randomly sampled across a 5 mm axial range, with each individual distance assigned a unique output label. This labeled dataset enables the RC model to learn the association between waveform features and specific depths. Figure 3 presents interference signal waveforms at 14 different distances, clearly demonstrating the strong dependence of temporal features on the axial distances. Due to the presence of nonlinear wavelength sweeping, frequency chirps have been evidenced in all the interference waveforms. These variations typically require complex nonlinear corrections when using Fourier-based analysis.

In this work, the RC model directly analyzes the temporal features in each waveform, capturing depth information efficiently without additional processing. By training on this diverse set of chirped signals, the RC model learns to classify depth-related patterns, making it a highly effective and computationally efficient solution for OCT depth extraction, even under challenging nonlinear wavelength sweeping conditions. This Fourier-free approach thus offers a simplified, powerful alternative for obtaining accurate depth information, making it well suited for real-time OCT applications.

Under such circumstances, using RC for temporal signal analysis proves to be an effective approach, as it eliminates the need for complex nonlinear frequency sweep correction. This method enables accurate extraction of depth information directly from the temporal interference signals, facilitating efficient signal processing and analysis in the context of varying fringe densities.

Following the training phase, we created a test dataset comprising 14 waveforms, each corresponding to a unique depth, as depicted in Figure 3. To ensure comprehensive evaluation, the test dataset includes four periods for each depth, where each period is represented by a data vector of length 500 × 1. Consequently, the total training set is made up of 56 samples, ensuring a robust model training process.

For testing, periods were randomly selected from the 14 distinct depths, ensuring that none of these specific periods overlapped with those in the training dataset. Instead, alternate periods matching the required length were used to construct the test dataset. By randomizing the sequence of periods, we aim to simulate real-world conditions and assess the RC model’s ability to generalize accurately to new data. This randomized order provides an additional layer of verification for the model’s depth classification performance, reinforcing its potential to maintain reliability even in variable conditions. Through this approach, the RC model’s robustness in interpreting time-domain OCT signals is validated, showcasing its effectiveness in reliably distinguishing depth without prior Fourier-based preprocessing, making it a strong candidate for real-time depth measurement applications in optical coherence tomography.

**Figure 4:**
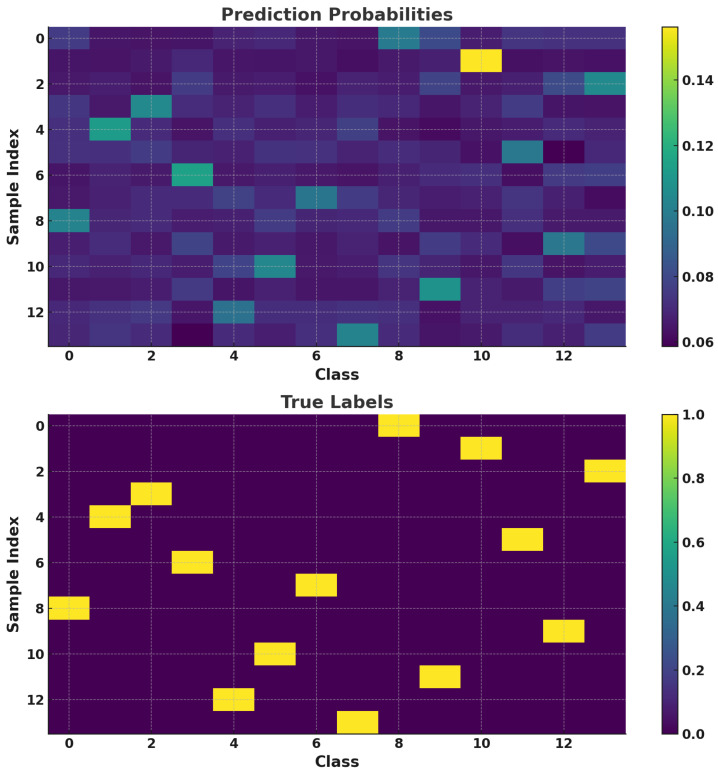
RC classification results of interference waveforms.

Figure 4 presents the testing results from the RC classification task. In the upper half of the figure, we see the model’s output represented by the probabilities of each output label. The lower half of the figure shows the true output labels indicating each sample’s actual depth. The RC model makes classification decisions based on the highest probability value within the output vector, where the sum of all output values equals 1, meaning that each individual value is relatively small but collectively adds up to a probability distribution. As shown in Figure 4, the RC model’s output labels align precisely with the actual labels across all 14 depth classes, achieving a classification accuracy of 100%. This high accuracy can be attributed to the controlled conditions under which the waveforms were recorded; all waveforms used for both training and testing were captured within a brief timeframe (20 ns per waveform). Consequently, environmental variations or system instability, which could otherwise introduce waveform distortion, was minimized. This stable setup allowed the RC model to classify each depth accurately, highlighting its effectiveness in a well-controlled environment. The results confirm the RC model’s potential for high-precision depth measurement in OCT systems, particularly when external factors are stable.

This probabilistic classification approach allows the RC model to perform accurate depth detection by assigning each signal to its most likely depth class. The model’s ability to produce distinct class probabilities enables a reliable depth classification, even in cases where the interference signal may have noise or minor distortions. Such a capability demonstrates the RC model’s robustness, validating its effectiveness in distinguishing between depths in OCT applications without requiring traditional Fourier-based analysis for signal processing.

**Figure 5:**
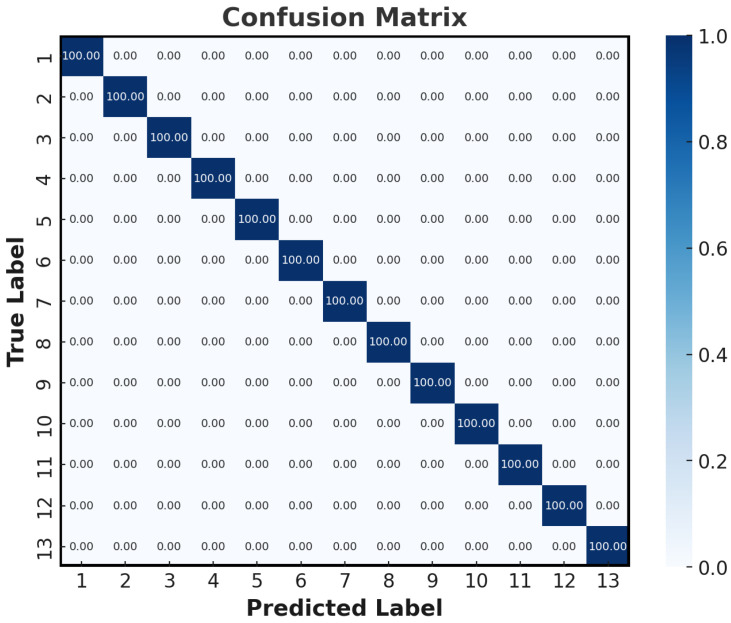
Identification results from RC.

To expand the dataset, we conducted 50 measurement cycles for each of the 13 different distance values, resulting in a total of 650 signal samples. Each of the 13 distance values was assigned a unique label, ranging from 1 to 13, to facilitate the classification task. For model training and testing, 80% of the dataset was allocated to training, while the remaining 20% was reserved for evaluating the classification accuracy of the RC system. The final test results, shown in Figure 5 via a confusion matrix, illustrate the system’s classification performance across various distances. The results reveal that the RC system effectively classified TS-OCT interference waveforms, achieving a 100% accuracy. This high level of performance demonstrates the robustness and precision of the RC model in depth measurement tasks, even under conditions involving multiple depth classifications. Such accuracy underscores the potential of RC-based approaches for real-time, high-resolution imaging in TS-OCT applications.

### 3.3. Waveform Classification Based on RC Model

To further evaluate the classification performance of the RC model on the captured interference signals, we conducted three sets of experiments. In each set, signals corresponding to 13 different depths were collected, and 4 periods of signals were selected within a 1 μs time window, resulting in a total of 156 samples used for training. To ensure robustness, the test set consisted of 52 periods from the 13 depths in a random order. These 52 periods were not selected from the training set but were instead other periods of the required length, randomly arranged to validate the reliability of the algorithm.

**Figure 6:**
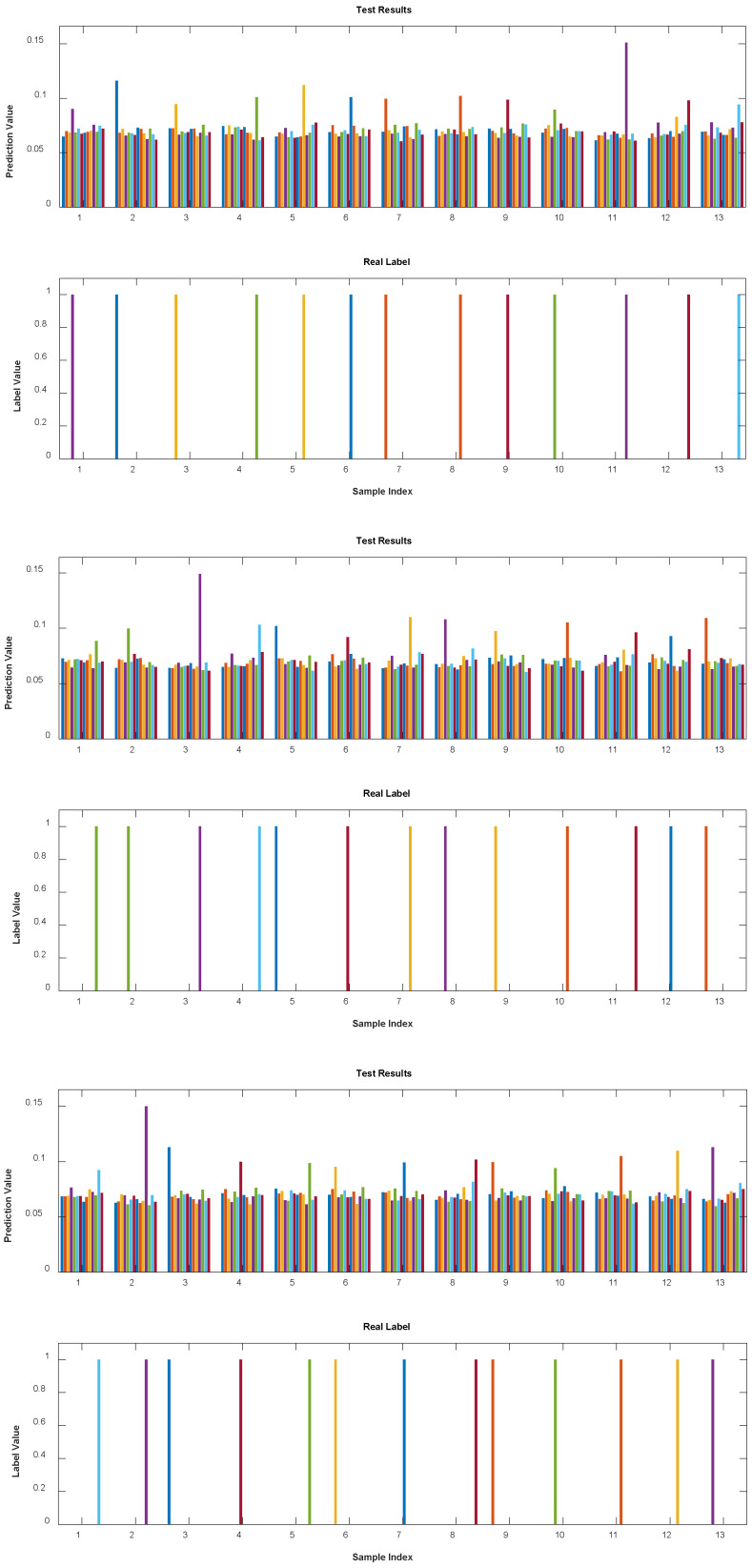
Classification results of the RC model for interference waveforms corresponding to 13 different depths from three sets of experiments. Different colors represent waveforms corresponding to different depths.

We conducted three experiments, each using different training and testing sets. We selected one of the classification results as shown in Figure 6. In each figure, the left half displays the test results, while the right half represents real labels at different distances, with different colors indicating different depths.

The experimental results demonstrated that the proposed RC model effectively identifies the interference waveforms corresponding to different target depths, achieving an overall accuracy of 100%. Furthermore, we assessed the classification performance of the RC model by calculating the root mean square error (RMSE) of the experimental results and comparing it with the RMSE of each training set. The results are summarized in Table 2 below. The average RMSE of the testing set is 0.2416, which indicates the strong classification capability of the RC model in accurately distinguishing interference signals from different depths.

### 3.4. Classify RC Using Different Datasets

**Figure 7:**
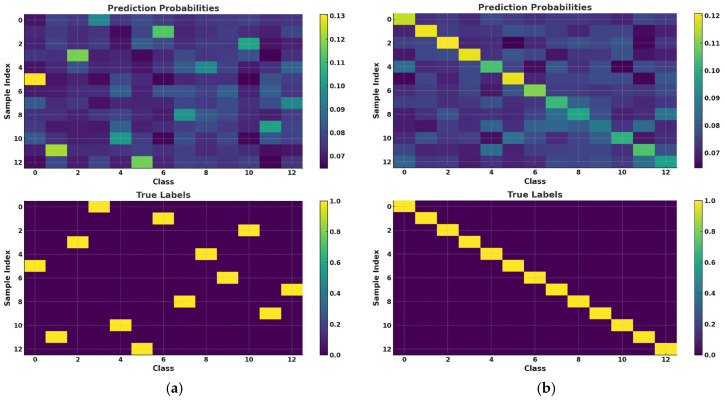

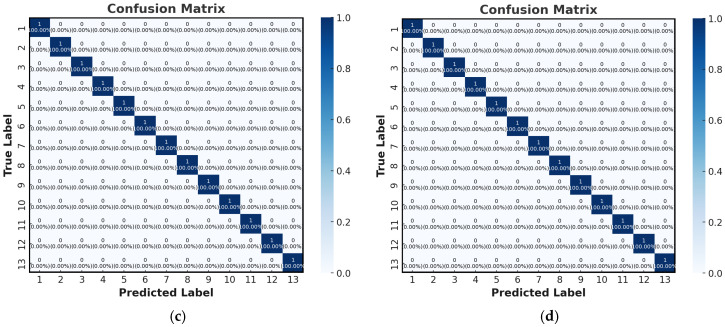
(**a**,**b**) are RC classification results of interference waveforms. (**c**,**d**) are identification results from RC via confusion matrix.

In the context of temporal signal waveform classification using reservoir computing (RC), we adopt a methodology that involves organizing labels in both sequential and randomized configurations, as shown in Figure 7a,b.

This approach allows for a more granular evaluation of the model’s sensitivity to temporal dependencies. A substantial performance improvement on sequentially ordered data relative to randomized data would suggest a strong dependence on temporal structures. In contrast, if the model maintains comparable performance on randomized data, it indicates a higher degree of independence in capturing global or local features beyond temporal sequencing.

Figure 7c,d illustrate two distinct representations of the confusion matrix for the same set of experimental results. In Figure 7c, the class labels are arranged sequentially, while in Figure 7d, the label order is randomized. Under the sequential arrangement of labels in Figure 7c, the deep blue regions along the diagonal of the confusion matrix signify that the model correctly classifies all samples, indicating perfect alignment between predicted and true values for each class, thereby achieving 100% classification accuracy. In contrast, when the label order is randomized, as shown in Figure 7d, the overall structure of the confusion matrix change s, but the model’s predictive performance remains consistent, demonstrating its robustness to variations in label ordering.

Furthermore, incorporating both sequential and randomized data enables the model to explore a broader feature space, thereby mitigating the risk of the model being overly reliant on a singular pattern, such as strict temporal dependencies. This diversification fosters a more robust generalization capability for the model when applied to unseen data.

Consequently, in real-world scenarios where the input time-domain waveforms may contain noise, exhibit missing segments, or experience perturbations in sequence, the model can still maintain a high level of classification accuracy.

Table 3 presents the RMSE of the model on both the training and test sets under different data arrangements: sequential and randomized. The table is divided into two columns, representing the RMSE results for sequential (Sequence) and randomized (Random) data arrangements. Although the RMSE for the sequential arrangement is slightly lower than that for the randomized arrangement, the difference is minimal, indicating that the data arrangement order has an insignificant impact on the model’s performance on the test set.

The RC model exhibits strong robustness and memory capacity when processing temporal data, resulting in low sensitivity to the arrangement order of the input data. The RMSE values for both sequential and randomized arrangements remain at comparable levels, demonstrating that the RC model can effectively capture the features of the input data without being substantially influenced by the order of data presentation.

According to the results in the table, the RC model maintains stable performance when handling both sequentially ordered and randomly ordered data. This stability arises from the unique structure of the reservoir network, which projects the dynamic characteristics of the input data into a high-dimensional space. As a result, the model can learn the intrinsic patterns of the data under varying input sequences, ensuring robust performance regardless of the order of the data arrangement.

### 3.5. Effect of Noise on the Robustness of the RC System

In TS-OCT systems, noise significantly impacts the robustness of classification models. Common noise sources include shot noise, detector noise, excess photon noise, and relative intensity noise (RIN). Shot noise arises from the discrete nature of photon or electron arrival events, which follow Poisson distribution. However, at higher light intensities, the Poisson distribution can be approximated by a normal distribution, making shot noise resemble Gaussian noise under such conditions. The root mean square (RMS) value of shot noise can be expressed as $i_{shot}=\sqrt{2qI∆f}$, where q represents the electron charge, I is the photocurrent, and ∆f is the measurement bandwidth. By introducing shot noise with varying intensities into the input signals, the model’s performance under low-light conditions can be systematically evaluated.

Detector noise includes thermal noise, dark current noise, and readout noise. Thermal noise, caused by the thermal motion of electrons within the detector, has an RMS value given by $V_{thermal}=\sqrt{4k_{B}TR∆f}$, where $k_{B}$ is the Boltzmann constant, T is the temperature, and R is the resistance. Dark current noise arises from the intrinsic current fluctuations present in the detector even in the absence of light, while readout noise originates from random fluctuations within the electronic readout circuitry. These noise sources are particularly impactful under low-light conditions, where their relative contributions are more pronounced. Simulating these different types of detector noise allows for a detailed assessment of their effects on the overall system noise and classification accuracy.

Excess photon noise predominantly occurs in coherent light sources, originating from mode competition or gain fluctuations within the laser cavity. This noise manifests as irregular intensity fluctuations, which can significantly interfere with the feature extraction process of RC models, especially when analyzing weakly reflective samples or deep tissue layers. By generating intensity signals with random amplitude and phase variations, the characteristics of excess photon noise can be effectively modeled, facilitating the evaluation of its impact on classification performance.

RIN results from random intensity fluctuations in the laser output, typically described by the power spectral density formula $RIN=\frac{∆I^{2}}{{\langle I\rangle }^{2}∆f}$, where ∆I denotes intensity fluctuations and $\langle I\rangle$ is the mean intensity. By adding RIN of varying intensities and frequency distributions to the signals, the model’s performance under dynamic intensity variations can be assessed. While the classification performance remains stable at low noise levels, it progressively deteriorates as noise intensity increases. At high noise levels, accuracy may approach that of random guessing, indicating the limits of the model’s robustness.

Overall, these noise sources can be approximated as Gaussian noise under certain conditions. This approximation is based on the Central Limit Theorem (CLT) in statistical theory, which states that when a random variable is the sum of many independent and identically distributed random variables, its distribution approaches a normal distribution, regardless of the original distribution of each variable. By systematically introducing and evaluating Gaussian noise, the robustness limits of RC models can be thoroughly analyzed, providing valuable insights and data support for improving the noise resilience and performance optimization of the models.

To evaluate the performance of the RC model under different noise levels, Gaussian noise of varying amplitudes was introduced into the test dataset. The addition of Gaussian noise simulates real-world stochastic interference, allowing an assessment of the RC model’s stability and accuracy in processing uncertain and noisy signals.

**Figure 8:**
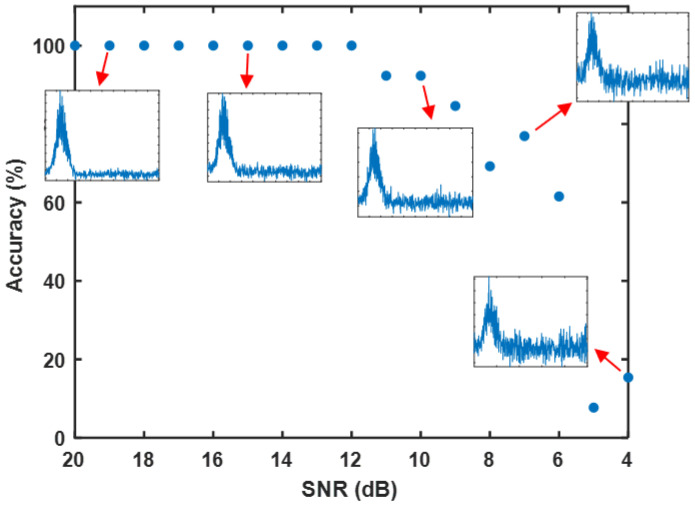
Classification accuracy as shown in blue dots vs. SNR of temporal signals with spectral insights at different noise levels.

As illustrated in Figure 8, the classification accuracy of the RC model was tested under various Gaussian noise amplitudes, quantified as the signal-to-noise ratio (SNR) in decibels (dB). The *x*-axis represents the SNR, ranging from 20 dB to 4 dB, while the *y*-axis indicates the classification accuracy in percentage. The results demonstrate a progressive decline in accuracy as the SNR decreases (i.e., as noise amplitude increases), underscoring the non-negligible impact of noise on signal quality and its substantial influence on model performance under lower SNR conditions.

The inset plots in Figure 8 highlight the spectral characteristics of the signals under different SNR levels, providing an intuitive understanding of the impact of noise. At high SNR values (e.g., 19 dB and 15 dB), the signal peaks are clearly discernible in the spectrum, with minimal noise interference. Under these conditions, the RC model effectively captures the signal features, achieving classification accuracies close to 100%. This observation indicates that the dynamic architecture of the RC model enables robust feature extraction from time-series signals under low-noise conditions, maintaining high classification performance.

However, as the SNR decreases to 11 dB and 7 dB, the spectral noise components become increasingly pronounced, making the signal features less distinguishable. At an SNR of 8 dB, the interference from noise on the primary signal peaks becomes apparent, leading to a noticeable drop in classification accuracy. This suggests that under moderate noise levels, the robustness of the RC model is challenged, as it struggles to extract sufficient distinguishing information from signals submerged in noise.

When the SNR is further reduced to 4 dB, the spectral noise nearly obscures the signal features entirely, resulting in a sharp decline in classification accuracy. At an SNR of 4 dB, the accuracy approaches zero, indicating that severe noise disrupts the signal structure to such an extent that the RC model fails to effectively recognize and classify the signals. This finding highlights the limitations of the RC model in handling high-noise environments, where its dynamic architecture becomes ineffective in preserving classification capabilities under severe signal distortion. Reducing the SNR below 4 dB is unnecessary, as the results already show that the RC model’s classification accuracy drops to near zero at this level. At 4 dB, noise completely dominates the signal, making further reductions redundant. If the system operates at even lower SNRs, pre-processing techniques such as using a balanced detector and applying filtering should be employed first to enhance the signal quality before classification.

In summary, the RC model exhibits a marked disparity in robustness when operating under varying noise levels. At high SNR values (greater than 15 dB), the model’s classification performance is virtually unaffected by noise, maintaining near-perfect accuracy due to its ability to efficiently extract relevant features from time-series data. Conversely, when the SNR falls below 11 dB, noise significantly impacts the signal feature extraction process. This effect becomes particularly severe at SNR values below 7 dB, where the model’s performance deteriorates sharply, reflecting its inherent limitations under high-noise conditions. These findings provide valuable insights into the RC model’s noise tolerance and highlight the need for potential enhancements to improve its resilience in noisy environments.

### 3.6. Maximum Resolution and Detection Depth of the RC-Based Fourier-Free System

The maximum resolution of the Fourier-free TS-OCT system is determined by the classification capability of the RC model when processing interference signals with minimal axial distance difference. The core principle of the RC model lies in projecting time-domain signals into a high-dimensional dynamic state space and mapping the signal features using fixed nonlinear activation functions. During this process, the RC state space amplifies differences in the input signals, enabling effective processing and classification of complex nonlinear signals.

However, when the depth axial distance difference is minimal, the interference signal waveforms in the time domain become very similar, leading to highly overlapping mapping results in the RC state space. This feature space overlap reduces the distinguishability between state characteristics, thus limiting the RC model’s classification capability for these signals.

Specifically, the classification capability of the RC model relies on the dynamic response features of the input signals in the state space. When the differences between input signals are insufficient to induce significant state changes, the RC output weight matrix cannot effectively classify the signals, resulting in classification errors. Therefore, the minimum resolution of the Fourier-transform-free TS-OCT system can be defined as the smallest depth difference that the RC model can reliably distinguish, i.e., the minimum input signal variation that can still form discernible features in the state space.

**Figure 9:**
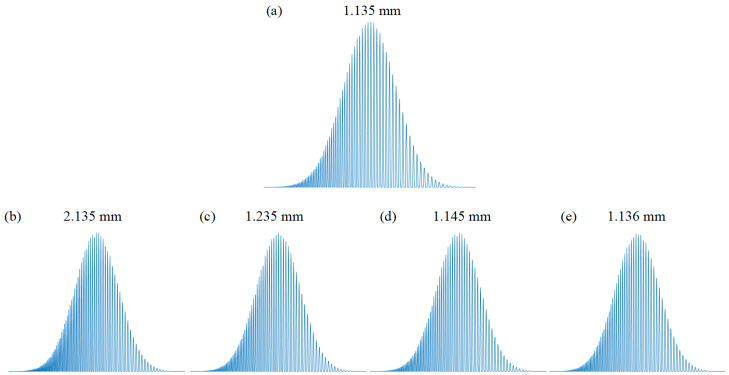
Simulated interference signal patterns corresponding to different axial depths: (**a**) 1.135 mm, showing a standard interference fringe pattern; (**b**) 2.135 mm, with a distinct change in fringe density; (**c**) 1.235 mm, demonstrating slightly denser fringes; (**d**) 1.145 mm, with minimal variation from the standard; and (**e**) 1.136 mm, highlighting the challenge of resolving closely spaced depths.

Numerical simulations were conducted to investigate the effect of interference signals at different depths on fringe density. Specifically, we performed a simulated analysis of interference signals for four different axial distance differences: 1 mm, 0.1 mm, 0.01 mm, and 1 μm, with the results shown in Figure 9. The simulation results indicate that as the axial distance difference decreases, the variation in fringe density of the interference signals becomes progressively weaker, imposing higher demands on the classification capability of the RC model.

To evaluate the RC model’s classification performance at different scales, we used simulated signals as inputs and conducted classification experiments. At scales of 1 mm, 0.1 mm, and 0.01 mm, the RC model successfully identified signals from different depths with 100% classification accuracy. However, at the ultra-small scale of 1 μm, the classification accuracy of the RC model dropped significantly to only 5.71%. This demonstrates that when the distance difference approaches the system’s resolution limit, the fringe density variation of the interference signals becomes insufficient to form separable features in the high-dimensional dynamic state space of the RC model, resulting in a substantial decline in classification performance.

To ensure the statistical reliability of the experimental results, we expanded the training and testing datasets by simulating 70 different distances at each scale. These signals were used for RC model training and testing, and the classification accuracy results are summarized in Table 4.

In OCT systems, the Fourier transform is widely used to convert frequency-domain data into depth-domain images. Therefore, the maximum detection depth of the system is fundamentally determined by the bandwidth of the PD and the oscilloscope. For this system, the oscilloscope’s bandwidth is 23 GHz, making it the primary limiting factor for the maximum detection depth.

Due to the presence of nonlinear dispersion effects in the system, the relationship between frequency and depth cannot be directly calculated. Instead, the Time Warp Theory is employed to accurately measure the connection between time and frequency, enabling the determination of the actual detection depth.

**Figure 10:**
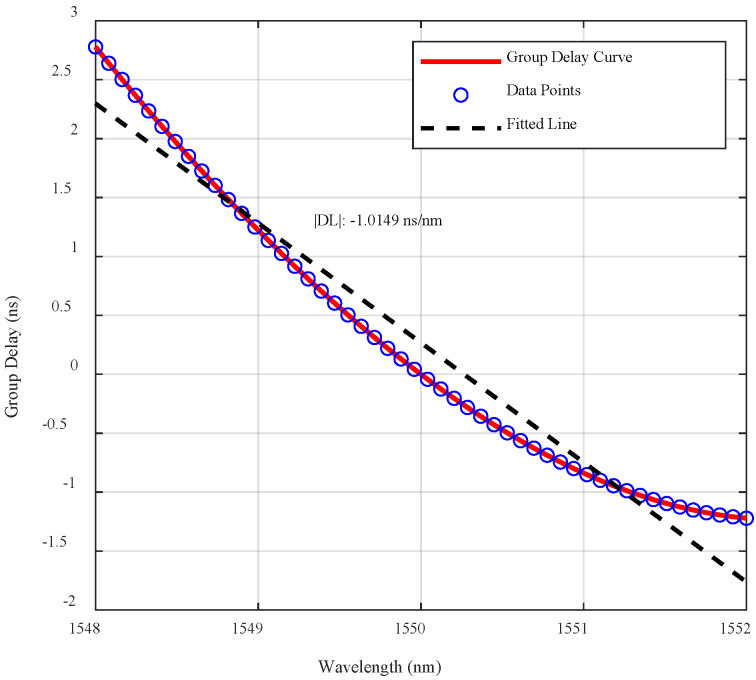
Group delay curve and fitted line for dispersion measurement.

The relationship between the pulse signal’s central wavelength and its time position is shown in Figure 10. By fitting the $t\left(\lambda \right)$ curve using a parabolic model, the following expression is obtained:

(4) $$t\left(\lambda \right)=t\left(\lambda_{0}\right)+LD\left(\lambda_{0}\right)\left(\lambda -\lambda_{0}\right)+LD^{\prime }{\left(\lambda -\lambda_{0}\right)}^{2}+LD^{\prime \prime }{\left(\lambda -\lambda_{0}\right)}^{3}$$

The formula for $t\left(\lambda \right)$ is a Taylor expansion around the reference wavelength $\lambda_{0}$, where $t\left(\lambda \right)$ represents a physical parameter (e.g., time delay) as a function of wavelength. The first term, $LD\left(\lambda_{0}\right)\left(\lambda -\lambda_{0}\right)$, indicates the value of this parameter at the reference wavelength $\lambda_{0}$. The second term, $LD^{\prime }{\left(\lambda -\lambda_{0}\right)}^{2}$, describes the linear variation when λ deviates from $\lambda_{0}$, while the third term, $LD^{\prime \prime }{\left(\lambda -\lambda_{0}\right)}^{3}$, reflects the nonlinear curvature near $\lambda_{0}$.

The relationship between the frequency shift ∆f of the Fourier transform peak and the actual depth is given by

(5) ∆f=1/ΔT

where Δ*T* is the pulse duration, determined by the spectral range ∆λ, system dispersion $D\left(\lambda \right)$, and fiber length *L*, as follows:

(6) ∆T=D(λ)LΔλ

This formula indicates that pulse broadening depends not only on the intrinsic properties of the dispersive medium but also on the spectral range and the medium’s length. By experimentally measuring Δ*T* and ∆λ, the system’s Free Spectral Range (FSR) can be calculated, and from the formula

(7) ∆λ=λ/2nl,

the Optical Path Difference (OPD), or the system’s maximum imaging depth *l*, is determined. Here, λ is the center wavelength, *n* is the refractive index, and *l* is the OPD. A quadratic fit of the dispersion curve provides the linear relation between wavelength and time, with |DL| = −1.015 ns/nm. Using the oscilloscope’s maximum bandwidth and the above formulas, the system’s maximum detection depth is calculated as *l* = 27.14 mm.

Experimental results show that within a depth difference range of 1 mm to 0.01 mm, the RC model exhibits excellent classification capability, achieving 100% accuracy in distinguishing interference signals at different depths. However, when the depth difference is reduced to the 1 μm level, the RC model’s classification performance significantly declines, with accuracy dropping below 10%. This indicates that under ultra-small depth differences, the feature differences between interference signals are too subtle to form separable features in the high-dimensional dynamic state space of the RC model, leading to reduced classification accuracy.

These findings validate the resolution limit of the RC model in the Fourier-transform-free TS-OCT system, defined as the minimum depth difference the RC can reliably distinguish. Furthermore, based on the system’s dispersion properties and Fourier transform relationships, the maximum detection depth of this Fourier-transform-free TS-OCT system is determined to be 27.14 mm, governed by the spectral range, dispersive medium properties, and oscilloscope bandwidth.

## 4. Conclusions

In this paper, we propose and demonstrate an innovative Fourier-free TS-OCT system that leverages RC for temporal signal processing. This system effectively addresses the primary challenges faced by traditional Fourier-based OCT methods, including signal distortion and reduced depth resolution caused by nonlinear frequency sweeping. By directly analyzing time-domain signals and utilizing the dynamic temporal processing capability of RC, the system achieves high-resolution depth measurement without relying on Fourier transform techniques.

Experimental results show that the RC-based system can accurately classify interference waveforms corresponding to different sample depths, achieving a classification accuracy of 100%. The system is capable of distinguishing depth variations at a scale as small as 0.01 mm, with a low RMSE. Compared with traditional Fourier-based OCT systems, the RC-based approach simplifies the data processing pipeline, reduces computational complexity, and eliminates the need for complex linearization and chirp compensation processes. This not only improves system efficiency but also enhances real-time signal processing capabilities, making it particularly suitable for high-speed imaging applications.

The system also demonstrates strong robustness and adaptability to variations in input data, ensuring stable performance across different datasets. Further robustness analysis was conducted by introducing varying levels of Gaussian noise to the test data. The results demonstrated that the RC model maintains high classification accuracy at higher SNR levels, with gradual performance degradation observed only at extremely low SNRs. This highlights the system’s ability to operate reliably in noisy environments, further extending its practical applicability. This capability makes the system well suited for applications in complex and dynamic environments where traditional methods may struggle. Furthermore, the relatively low computational requirements of the RC model enable effective handling of large datasets, positioning it as a promising alternative for real-time imaging and signal classification in biomedical and industrial environments. Moreover, experimental results show that in the range of axial depth differences from 1 mm to 0.01 mm, the RC model exhibits excellent classification capability, achieving 100% accuracy in distinguishing interference signals at these scales. However, as the depth difference reduces to the scale of 1 μm, the RC model’s classification performance drops significantly, with accuracy falling below 10%. This limitation is due to the insufficient distinction between interference signal patterns at extremely small depth differences, reflecting the resolution limit of the RC model. At the same time, the maximum detectable depth of the system, calculated using dispersion characteristics and the oscilloscope bandwidth, is 27.14 mm. This result highlights the robustness of the RC model over a wide depth range and its constraints in resolving ultra-small depth differences.

Future work can explore optimizing the RC model architecture and training procedures to further enhance performance. Additionally, integrating this approach with other photonic signal processing techniques could lead to the development of more advanced optical imaging systems. Potential applications include non-invasive medical diagnostics, high-speed industrial inspection, and environmental monitoring, where precise imaging is critical.

## Figures and Tables

**Table 1:** Comparative performances of RC, RNNs, and LSTMs in TS-OCT.

	Classification Accuracy (%)	Computational Complexity	Training Time (s)
RC	100%	O(N)	9
RNN	94%	$O(N^{2})$	116
LSTM	96%	$O(N^{2})$	175

**Table 2:** The RMSE of classification results.

	Experiment 1	Experiment 2	Experiment 3
RMSE for Testing Sets	0.241178	0.242061	0.241625
RMSE for Training Sets	0.239507	0.239518	0.239424

**Table 3:** The RMSE of sequential and randomized classification results.

	Sequence	Random
RMSE for Testing Sets	0.257623	0.258119
RMSE for Training Sets	0.249171	0.249102

**Table 4:** Classification accuracy of the RC model at different axial distance scales.

Axial distance difference	1 mm	0.1 mm	0.01 mm	1 μm
Classification accuracy (%)	100.00	100.00	100.00	5.71

## Data Availability

Data are contained within the article.
